# Berbamine Analogs Exhibit Differential Protective Effects From Aminoglycoside-Induced Hair Cell Death

**DOI:** 10.3389/fncel.2020.00234

**Published:** 2020-07-29

**Authors:** Alexandria M. Hudson, Gavin M. Lockard, Ojas A. Namjoshi, Joseph W. Wilson, Katie S. Kindt, Bruce E. Blough, Allison B. Coffin

**Affiliations:** ^1^Integrative Physiology and Neuroscience, Washington State University, Vancouver, WA, United States; ^2^College of Arts and Sciences, Washington State University, Vancouver, WA, United States; ^3^RTI International, Research Triangle Park, NC, United States; ^4^National Institute on Deafness and Other Communication Disorders, National Institutes of Health, Bethesda, MD, United States

**Keywords:** hair cell, aminoglycoside, zebrafish, lateral line, berbamine, ototoxicity, hearing loss, mechanotransduction

## Abstract

Hearing loss is the third most common chronic health condition in the United States and largely results from damage to sensory hair cells. Major causes of hair cell damage include aging, noise exposure, and medications such as aminoglycoside antibiotics. Due to their potent antibacterial properties and low cost, aminoglycosides are often used for the treatment of gram-negative bacterial infections, surpassing expensive antibiotics with fewer harmful side effects. However, their use is coupled with permanent hearing loss in over 20% of patients requiring these life-sustaining antibiotics. There are currently no FDA-approved drugs that prevent hearing loss from aminoglycosides. A previous study by our group identified the plant alkaloid berbamine as a strong protectant of zebrafish lateral line hair cells from aminoglycoside damage. This effect is likely due to a block of the mechanotransduction channel, thereby reducing aminoglycoside entry into hair cells. The present study builds on this previous work, investigating 16 synthetic berbamine analogs to determine the core structure underlying their protective mechanisms. We demonstrate that nearly all of these berbamine analogs robustly protect lateral line hair cells from ototoxic damage, with ED_50_ values nearing 20 nM for the most potent analogs. Of the 16 analogs tested, nine strongly protected hair cells from both neomycin and gentamicin damage, while one conferred strong protection only from gentamicin. These data are consistent with prior research demonstrating that different aminoglycosides activate somewhat distinct mechanisms of damage. Regardless of the mechanism, protection required the entire berbamine scaffold. Phenolic alkylation or acylation with lipophilic groups appeared to improve protection compared to berbamine, implying that these structures may be responsible for mitigating damage. While the majority of analogs confer protection by blocking aminoglycoside uptake, 18% of our analogs also confer protection *via* an uptake-independent mechanism; these analogs exhibited protection when delivered after aminoglycoside removal. Based on our studies, berbamine analogs represent a promising tool to further understand the pathology of aminoglycoside-induced hearing loss and can serve as lead compounds to develop otoprotective drugs.

## Introduction

Sensory hair cells can be damaged from aging, intense noise exposure, and medications such as aminoglycoside antibiotics. In mammals, damaged hair cells are not replaced, which can result in permanent hearing loss. In humans, hearing loss can be devastating since it can lead to social isolation and decreased employment opportunities (Raviv et al., 2010; Jung and Bhattacharyya, 2012; Bainbridge and Wallhagen, 2014; Mick et al., 2014). Despite the adverse effects of aminoglycosides on hearing, they are widely used in developing nations due to their potent antibacterial properties and low cost, surpassing expensive antibiotics with fewer harmful side effects. Currently, there are no FDA-approved drugs that prevent aminoglycoside-induced hearing loss. Our work seeks to develop a drug therapy that robustly prevents aminoglycoside-induced hearing loss by modifying the chemical scaffold of a naturally occurring otoprotective compound.

There is strong evidence supporting mechanoelectrical transduction (MET) as the primary path for aminoglycosides to enter hair cells. The MET channels in hair cells are non-selective cation channels with a pore diameter of 1.25 nm. These features enable relatively large positively charged aminoglycosides to permeate the channel (Farris et al., 2004). Additionally, there is a membrane potential difference between the extracellular fluid and the negatively polarized cytoplasm that increases cellular uptake of positively charged aminoglycosides (Marcotti et al., 2005; Myrdal and Steyger, 2005). Pharmacologically blocking the MET channel dramatically decreases aminoglycoside uptake (Marcotti et al., 2005) further supporting antibiotic entry *via* this route. In addition to MET channels, there are also secondary entry routes occurring *via* endocytosis or through other ion channels (Portmann et al., 1974; Myrdal and Steyger, 2005; Karasawa et al., 2008; Hailey et al., 2017). The current hypothesis surrounding entry *via* endocytosis is that aminoglycosides are initially sequestered by endosomes, then trafficked to lysosomes, but different aminoglycosides (e.g., neomycin vs. gentamicin) differ in their rates of uptake into subcellular compartments. These data imply that sequestration of aminoglycosides in lysosomes could potentially attenuate hair cell damage (Hailey et al., 2017). Regardless of the entry route, aminoglycosides accumulate in hair cells, leading to pathological consequences.

In light of our understanding of the mechanisms of aminoglycoside toxicity, new targets for protection are arising. Given that the MET channel is the primary entry route for aminoglycosides, one option for protection is to block entry of aminoglycosides through the channel. Prior work using a zebrafish lateral line assay identified two such compounds, PROTO-1 and PROTO-2, both of which protected hair cells from neomycin toxicity (Owens et al., 2008). Optimization of PROTO-1 yielded ORC-13661, an otoprotective lead compound that acts as a permeant MET channel blocker (Owens et al., 2008; Chowdhury et al., 2018; Kitcher et al., 2019). In a separate study, Kenyon et al. (2017) used zebrafish to identify an N-methyl-D-aspartate (NMDA) receptor antagonist and a selective potassium channel antagonist that also protected hair cells by attenuating aminoglycoside entry. Here, we use a zebrafish lateral line assay to assess the relative protection conferred from a modified scaffold of an otoprotective plant alkaloid. Our modifications are designed to diversify the alkaloid’s pharmacological activity to modulate multiple aspects of hair cell death, leading to a stronger therapy.

A previous study by our lab screened 502 natural compounds using a zebrafish model for ototoxicity and identified four otoprotective bisbenzylisoquinoline analogs: berbamine, E6 berbamine, hernandezine, and isotetrandrine, with berbamine being the most protective (Kruger et al., 2016). These analogs share a macrocyclic bistetrahydroisoquinoline ring scaffold and robustly protect hair cells from aminoglycoside damage, likely by attenuating aminoglycoside entry. These data are consistent with Ou et al. (2009, 2012), who demonstrated that quinoline ring compounds such as tacrine and chloroquine reduce aminoglycoside uptake by hair cells, leading to increased hair cell survival. Berbamine also reduces aminoglycoside-induced hair cell death in mice, likely by reducing aminoglycoside loading into the cochlea (Kirkwood et al., 2017). However, high concentrations of berbamine (30 μM) were toxic to murine cochlear hair cells. Screening additional berbamine analogs offer an excellent opportunity to identify moieties that are responsible for berbamine’s protective activity while avoiding the toxicity seen at high concentrations. This information will allow us to develop a non-toxic compound that maintains the diverse and protective pharmacological properties of berbamine to develop a robust therapeutic compound.

Bisbenzylisoquinoline compounds have recently attracted attention as therapeutics due to their innate chemical diversity and wide-ranging pharmacological effects, making them powerful scaffolds for drug optimization (Tian and Zheng, 2017; Teles et al., 2019). Berbamine, a plant alkaloid derived from Berberidaceae (barberry plants), has its pharmacological origins in traditional Chinese medicine as an anti-inflammatory agent. More recently, berbamine has shown potential as a therapeutic to reduce inflammation from a diverse array of cancers, thus inhibiting tumor cell invasion (Ren et al., 2008; Liang et al., 2016; Jia et al., 2017). Here, we investigate the protective role of berbamine analogs in a zebrafish model of aminoglycoside-induced hair cell death. The study of structural analogs offers the opportunity to identify structural moieties of berbamine that may be responsible for its protective activity and enable the development of a robust and targeted therapeutic, the long-term goal of this work.

The zebrafish has become an increasingly popular vertebrate model in biomedical research, in part due to their small transparent larvae that are produced in large clutches, providing an excellent system for high-throughput drug screening (Ou et al., 2009, 2010; Coffin et al., 2010; Esterberg et al., 2013; Kenyon et al., 2017). Zebrafish and other fishes possess a sensory system known as the lateral line which is composed of hair cells clustered into groups (neuromasts). These hair cells are structurally and functionally similar to mammalian hair cells and show similar responses to ototoxic damage. The lateral line functions to detect vibrations in the water and to initiate an appropriate behavioral response such as predator avoidance or schooling. One advantage of using this model is that the hair cells are located externally, allowing for easy pharmacological manipulation *in vivo*. Using this advantage, chemical genetic screens have been performed to identify compounds that protect against aminoglycoside-induced hair cell loss. To date, the zebrafish lateral line has served as a platform for the discovery of several drug candidates with otoprotective potentials, such as PROTO- 1 (the precursor to ORC-13661), berbamine, tacrine, afimoxifene, and linopirdine (e.g., Ton and Parng, 2005; Owens et al., 2008; Ou et al., 2009; Vlasits et al., 2012; Kruger et al., 2016; Kenyon et al., 2017). Furthermore, some of these drugs confer similar protections in the mammalian inner ear (Ou et al., 2009; Kenyon et al., 2017; Kirkwood et al., 2017; Majumder et al., 2017; Chowdhury et al., 2018; Kitcher et al., 2019; O’Reilly et al., 2019).

Here, we report the synthesis and study of 16 berbamine analogs as potential otoprotective therapeutics. We demonstrate that alkylated and acylated berbamine analogs attenuate aminoglycoside-induced hair cell death in the zebrafish lateral line. Tetrahydroisoquinoline monomers were inactive and failed to attenuate cell death. The majority of active analogs confer protection by attenuating aminoglycoside entry into hair cells. Interestingly, a subset of analogs also confer protection *via* an uptake-independent mechanism. The compounds that show a multimodal mechanism of protection may be stronger leads for developing a therapy because they can potentially modulate multiple targets to inhibit hair cell death. This work demonstrates the potential clinical effectiveness of berbamine analogs as a therapeutic to prevent aminoglycoside ototoxicity.

## Materials and Methods

### Compound Synthesis

All solvents and chemicals for compound synthesis were purchased from Acros Organics, Sigma-Aldrich, Combi-Blocks, or AstaTech, Inc. and were used as received. All evaporations were carried out in vacuo with a rotary evaporator. Nuclear magnetic resonance spectra for proton (^1^H NMR) were recorded on a Bruker 300 MHz NMR spectrometer. The chemical shift values are expressed in ppm (parts per million) relative to tetramethylsilane as an internal standard: s, singlet; d, doublet; t, triplet; q, quartet; m, multiplet; br, broad singlet. The relative integrals of peak areas agreed with those expected for the assigned structures. Elemental analyses were performed by AtlanticMicrolab, Incorporation (Norcross, GA, USA). Element compositions are within 0.4% of the calculated values. Thin-layer chromatography (TLC) was performed on WHATMAN UV254 silica gel plates with a fluorescent indicator and the spots were visualized under 254 and/or 365 nm illumination. Flash chromatography was performed on a Teledyne ISCO CombiFlash chromatography system using pre-packed silica gel columns purchased from Teledyne ISCO.

### Animal Husbandry

All experiments were approved by the Institutional Animal Care and Use Committee at Washington State University (protocol # 6024) or by the Animal Care and Use Committee at the National Institutes of Health (NIH; protocol #1362–13). Zebrafish *(Danio rerio)* were maintained in aquatic facilities at Washington State University Vancouver or the NIH (calcium imaging experiments only). Zebrafish embryos were obtained by breeding pairs of adults and raising the embryos at 28°C. Unless stated otherwise, all experiments were conducted using E2 Embryo Medium (EM; 1 mM MgSO_4_, 120 μM KH_2_PO_4_, 74 μM Na_2_HPO_4_, 1 mM CaCl_2_, 500 μM KCl, 15 mM NaCl_2_ in distilled water at a pH of 7.2; Westerfield, 2000). Larval zebrafish between 5–6 days post-fertilization (dpf) in the wildtype *AB strain background were used for experiments unless specifically noted. *Tg(myo6b:EGFP), Tg(Brn3c:GFP)* or *Tg(α-tubulin: tdTomato)* transgenic lines were used to visualize hair cells for a subset of experiments. We selected fish between 5–6 dpf because their hair cells show adult-like responses to ototoxic damage (Murakami et al., 2003; Santos et al., 2006). For calcium imaging experiments to detect mechanosensitive responses in lateral line hair bundles, the previously described transgenic line was used: *Tg(-6myo6b:GCaMP6s-CAAX)^idc1Tg^* (Zhang et al., 2018). This line was maintained in the wildtype Tübingen strain background.

### Berbamine Analog Dose-Response

To create a dose-response curve, zebrafish (in 6-well plates) were pretreated with varying log and half-log concentrations (0.0032 μM to 9.98 μM) of a berbamine analog for one hour before aminoglycoside treatment (neomycin or gentamicin, both from Sigma-Aldrich, St. Louis, MO, USA, N1142 and G1397, respectively). Analogs were reconstituted in dimethylsulfoxide (DMSO) and diluted in EM before zebrafish administration. The dilutions were controlled so the final % of DMSO was the same across all analogs (0.5%). Control fish were treated with DMSO (vehicle) in the absence of analog. Larval zebrafish were exposed to one of two aminoglycoside treatment paradigms, defined here as either “acute” or “chronic” exposure. For acute treatment, zebrafish were exposed to 200 μM neomycin for 30 min in the presence of an analog, followed by four rinses in EM and a 60-min recovery in EM with no drugs present. Chronic exposure consisted of 100 μM gentamicin for 6 h followed by four rinses in EM and a 15-min recovery. We selected these aminoglycosides and exposure paradigms because they both induce hair cell damage with distinct time courses and mechanisms (Owens et al., 2009; Coffin et al., 2013a,b).

### Hair Cell Assessment

The majority of our hair cell assessment was done using 2-(4-(dimethylamino)styryl)-N-ethylpyridinium iodide (DASPEI; ThermoFisher, Eugene, OR, USA, D426), a mitochondrial dye that robustly labels the hair cells in the lateral line of zebrafish and other fishes (Harris et al., 2003; Coffin et al., 2009; Brown et al., 2010). After aminoglycoside and/or analog treatments, zebrafish were incubated for 15 min at 28°C with 0.005% DASPEI. The fish were subsequently rinsed 2× with EM and anesthetized with 0.001% buffered tricaine methanesulfonate (MS-222, Syndel, Ferndale, WA, USA) before visualization. We assessed the relative fluorescence intensity for 10 head neuromasts on every zebrafish (IO1, IO2, IO3, IO4, M2, O2, MI1, MI2, SO1, SO2; Raible and Kruse, 2000) using 50x magnification on a Leica M165F fluorescent dissecting microscope. Each neuromast was given a score to quantify the fluorescence: 2 (bright labeling), 1 (modest labeling), and 0 (no labeling); the individual neuromast scores were added to provide an overall score between 0–20 per fish (Harris et al., 2003; Coffin et al., 2009; Owens et al., 2009; Kruger et al., 2016). Scores were averaged per treatment group. To normalize the data, all treatment group averages were divided by the average value of the controls for that experiment and multiplied by 100 to arrive at the percentage of surviving hair cells. DASPEI labeling is a viable method of assessing hair cells with comparable results to directly counting immunolabeled hair cells (Harris et al., 2003; Coffin et al., 2013a; Kruger et al., 2016). For a subset of experiments, we used *Tg(Brn3c:GFP)* larvae, where hair cells express membrane-specific GFP. These experiments provided observation of cellular morphology following aminoglycoside and analog treatment. Animals were treated as described above for analog and aminoglycoside exposure, then euthanized with 0.002% buffered MS-222, fixed in 4% paraformaldehyde, and imaged on a Leica SP8 confocal microscope.

### Protection After 24 h

To determine whether we were observing true protection of hair cells from aminoglycoside damage rather than a delay in hair cell death, we exposed zebrafish to the optimal protective concentration (OPC) of each of our analogs for 1 h followed by a co-treatment with analog and 200 μM gentamicin for 30 min, with a 24-h recovery. This time course was selected to examine the protective capacity of our analogs in a prolonged phase of aminoglycoside exposure since gentamicin-induced hair cell damage continues after drug removal (Owens et al., 2009). Zebrafish were then rinsed 4× in EM and anesthetized with MS-222 before DASPEI labeling and hair cell assessment.

### GTTR Loading Assay

To quantify the uptake of aminoglycosides in the presence of berbamine analog, we exposed our zebrafish to gentamicin conjugated with Texas Red (GTTR), which predominantly enters hair cells *via* the MET channel (Steyger et al., 2003; Alharazneh et al., 2011). Zebrafish were first administered a one-hour pretreatment of the optimally protective concentration of analog followed by co-treatment with analog and 50 μM GTTR (made according to Steyger et al., 2003) for 18 min (Steyger et al., 2003; Alharazneh et al., 2011; Kruger et al., 2016). The fish were then rinsed 4× in EM before euthanasia with 0.002% buffered MS-222 and fixation in 4% paraformaldehyde, then imaged on a Leica SP8 confocal microscope using a 20× dry objective with 5× digital zoom. The gain and laser settings were constant across all groups. The image stacks were collapsed into maximum projection images before image analysis *via* ImageJ. The images were analyzed by subtracting the background and measuring the fluorescence intensity (arbitrary units; a.u.) of each neuromast and taking an overall average per group (Kruger et al., 2016). The IO2 neuromast was selected for consistency throughout the experiments.

### Washout Experiment

To further understand the mechanism(s) of protection by berbamine analogs, we exposed zebrafish to one of three treatment paradigms that varied in the timing of aminoglycoside administration relative to berbamine analog administration. The first paradigm relied on a pre-co treatment where fish were exposed to the analog both before and during gentamicin exposure. In the second paradigm, there was a co-treatment of the analog and aminoglycoside only during gentamicin exposure. In the third paradigm, post-treatment, the analog was applied after gentamicin removal. In all three treatments, we used a 30-min exposure to 200 μM gentamicin followed by a 5.5-h recovery in EM or (for the post-treatment time course) berbamine analog (Kruger et al., 2016). This aminoglycoside time course was selected since gentamicin-induced hair cell damage continues after ototoxin removal, allowing us to assess the optimal protective paradigm in a slower phase of hair cell death (Owens et al., 2009). Zebrafish were then rinsed 4× in EM and anesthetized with MS-222 before the DASPEI hair cell assessment.

### Hair Cell Viability Assessment

To confirm that our analogs were not compromising the viability of the hair cells or the function of the MET channel after prolonged analog exposure and subsequent removal, we assessed the hair cells using a combination of two vital dyes, YO-PRO-1 and FM 1–43FX (both from ThermoFisher Scientific, Eugene, OR, USA, Y3603 and F35355, respectively). YO-PRO-1 is a nucleic acid stain that specifically labels lateral line hair cells in live larvae, allowing us to visualize nuclear morphology (Santos et al., 2006). It is also a nontoxic dye that can be retained for 24 h making it suitable for long-term hair cell assessments (Thomas et al., 2015; Kruger et al., 2016; Neveux et al., 2016). FM1–43FX is a membrane probe that readily enters hair cells *via* the MET channel (Gale et al., 2001; Corey et al., 2004; Alharazneh et al., 2011) making it a good proxy for MET channel function following analog removal. Zebrafish were exposed to 3 μM YO-PRO-1 for 1 h followed by three rinses in EM. After labeling, the zebrafish were then incubated with analog for 24 h, rinsed 4× with EM, and left to recover for an hour to allow for dissociation of the analog from the MET channel (e.g., Kirkwood et al., 2017). Zebrafish were then incubated with 1.5 μM FM1–43FX for 30 s, rinsed 4× in EM, and anesthetized with MS-222 before live imaging on a Leica SP8 confocal microscope. All imaging was done sequentially on live anesthetized fish to resolve discrete signals between the fluorophores since FM1–43FX has a broad emission spectrum that overlaps with YO-PRO-1. In a separate experiment, *Tg(myo6b:EGFP)* zebrafish also were used to confirm that berbamine analogs are not ototoxic. We exposed our *Tg(myo6b:EGFP)* zebrafish to the OPC of our analogs for 24 h followed by 4× rinses in EM. The zebrafish were then euthanized with MS-222 followed by fixation with 4% paraformaldehyde before confocal imaging of neuromast IO2 for all zebrafish. The gain was kept constant across all groups.

### Fluorescent Analog Localization

We examined a fluorescently tagged berbamine analog to determine if analogs can enter hair cells. We used *Tg(α-tubulin: tdTomato)* zebrafish (to visualize hair cells) and pre-treated them with a fluorescent 7-(diethylamino)coumarin-tagged berbamine analog (BA-17) for one hour in EM before confocal imaging. The absorption and emission maxima of the coumarin tag are 409 nm and 473 nm, respectively. To test that the fluorescent tag did not interfere with the protective effects of the analog, we pretreated zebrafish for one hour with the fluorescently tagged analog before acute neomycin exposure. Zebrafish were then rinsed 4× with EM and anesthetized with MS-222 before DASPEI labeling and hair cell assessment. To examine if the punctate labeling from BA-17 colocalized with lysosomes, we used AB zebrafish labeled with LysoTracker (ThermoFisher, Eugene, OR, USA, L7528). Zebrafish were pretreated with BA-17 for 1 h followed by a 3-min exposure to 50 nM LysoTracker before confocal imaging of the IO2 neuromast.

### Functional Calcium Imaging in Zebrafish Hair Bundles

The protocol to image GCaMP6s-based calcium signals in zebrafish hair bundles has been described previously in detail (Lukasz and Kindt, 2018). Briefly, before imaging, individual larvae were anesthetized with 0.03% MS-222 (Sigma) and then pinned onto a Sylgard-filled recording chamber. To suppress the movement, alpha-bungarotoxin (125 μM, Tocris, Minneapolis, MN, USA) was injected into the heart. Larvae were then immersed in extracellular imaging solution (in mM: 140 NaCl, 2 KCl, 2 CaCl_2_, 1 MgCl_2,_ and 10 HEPES, pH 7.3) without MS-222. A fluid-jet (a glass pipette connected to an HSPC-1 device, ALA Scientific, Farmingdale, NY, USA) was used to apply a 500-ms anterior or posterior stimulus to deflect the apical bundles of trunk neuromasts (L1-L4). After baseline GCaMP6s responses were recorded, the extracellular imaging solution was exchanged with the same solution containing a berbamine analog. The solution in the fluid-jet pipette was also exchanged. Ten minutes after the solutions were exchanged, the same hair bundles were restimulated and the GCaMP6s responses were reexamined. Calcium imaging was performed blind concerning the protective capacity of each berbamine analog.

To capture calcium-dependent GCaMP6s signals in apical hair bundles, we used a Bruker Swept-field confocal system equipped with a Rolera EM-C2 CCD camera (QImaging) and a Nikon CFI Fluor 60× 1.0 NA water immersion objective. To coordinate stimulation with image acquisition, the fluid-jet was driven by a voltage-step command from the imaging software (Prairie view) during image acquisition. To simultaneously image the calcium signals in all hair bundles a piezoelectric motor (PICMA P-882.11–888.11 series, PI instruments, Auburn, MA, USA) attached to the objective was used to allow rapid imaging in five planes along the Z-axis at 0.5 μm intervals. Z-stacks were acquired at a 50 Hz frame rate yielding a 10 Hz volume rate. Calcium imaging experiments were performed on a minimum of four animals and seven neuromasts on two independent days. For image processing and quantification, the five plane Z-stack at each timepoint was projected into a single plane. The GCaMP6s signals in the projected images were then measured in FIJI. In FIJI, a circular region of interest with a ~1.5 μm diameter was placed on the center of each bundle and a peak finding program was used to determine the ΔF/F_0_ for each bundle. We quantified both the average bundle ΔF/F_0_ per neuromast and the temporal representation of the average ΔF/F_0_ signals.

### Data Analysis

Data were analyzed *via* one-way ANOVA using GraphPad Prism version 8. *Post hoc* comparisons were performed using Bonferroni or Tukey corrections for multiple comparisons (see figure legends for details). For calcium imaging, statistical differences in hair bundle calcium responses were determined using a paired, two-tailed Student’s *t*-test. Data are presented as mean ± SEM. ED_50_ analysis was conducted by normalizing all *y-values* and analyzing dose-response stimulus for agonist concentration vs. normalized response using GraphPad Prism version 8.

## Results

### Berbamine Analog Synthesis

**Figure 1:**
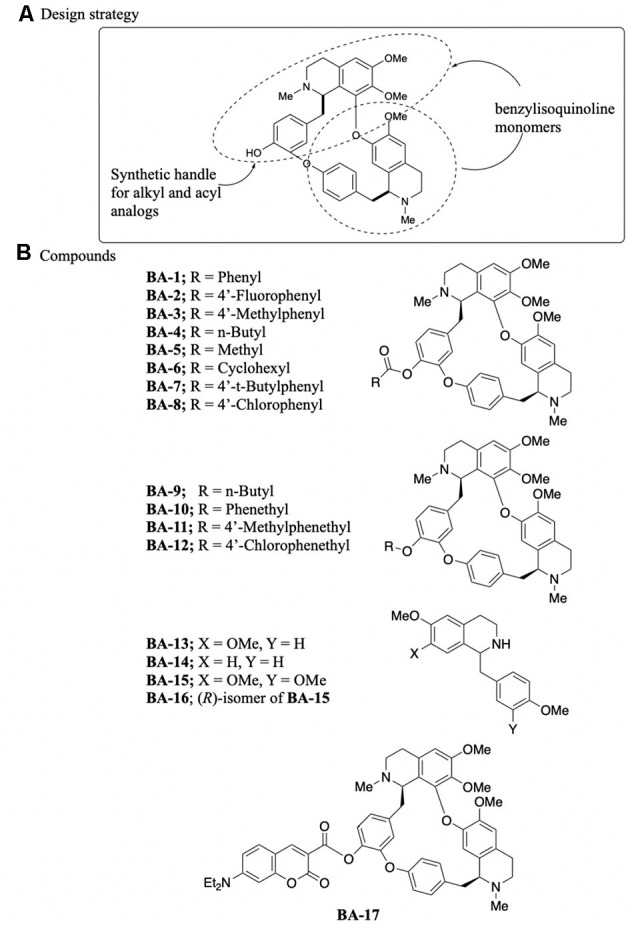
Synthesis of berbamine analogs. **(A)** Design strategy, including the synthetic handle. **(B)** Specific analogs synthesized for otoprotection studies. BA-13 through BA-16 are monomers, while BA-17 is a fluorescent analog.

Berbamine is a bisbenzylisoquinoline comprised of two structurally different benzyltetrahydroisoquinolines in which the benzyl groups are linked on one end and the isoquinoline groups are linked on the other, forming a macrocycle (Figure 1A). We selected an analog synthesis strategy based on the chemistry of the parent berbamine compound, to explore alkyl and phenyl substitutions and the contribution of the dual ring structure to otoprotective activity. Berbamine has one readily available synthetic handle, the phenol, which was used to synthesize our analogs (Figure 1A). This synthetic strategy maintained both the O-acylated (E6 berbamine) and O-alkylated (isotetrandrine) version of berbamine, based on our prior data demonstrating that both of these structures confer otoprotection (Kruger et al., 2016). Eight acylated berbamine analogs were synthesized as shown in Figure 1B (BA-1 to BA-8). Analogs were synthesized using the Steglich esterification method by reacting berbamine with a carboxylic acid in the presence of N,N^′^-dicyclohexylcarbodiimide and 4-dimethylaminopyridine. Four alkylated berbamine analogs were synthesized (BA-9 to BA-12). These were synthesized using the standard Williamson etherification conditions by reacting berbamine with sodium hydride, and then alkylating the intermediate phenoxide anion with an alkyl bromide in the presence of a catalytic amount of sodium iodide. These two approaches enable the study of various analogs modified on the phenol position.

Four benzyltetrahydroisoquinolines (BA-13 to B-16) were also studied and considered monomers of the bisbenzylisoquinoline ring system. BA-15 and BA-16 were commercially available. BA-13 and BA-14 were synthesized using a slightly modified literature procedure based on Perrey et al. (2013). 4-Methoxyphenylacetic acid was converted into amides with either 4-methoxyphenethylamine or 3, 4-dimethoxyphenethylamine using propanephosphonic acid anhydride as a coupling reagent and Hünig’s base. The resulting amides were then cyclized into intermediate dihydroisoquinolines using phosphorous oxychloride in toluene at 90°C followed by reduction of the resulting imine with sodium borohydride to form the final compounds.

### Berbamine Analogs Confer Dose-Dependent Protection

**Figure 2:**
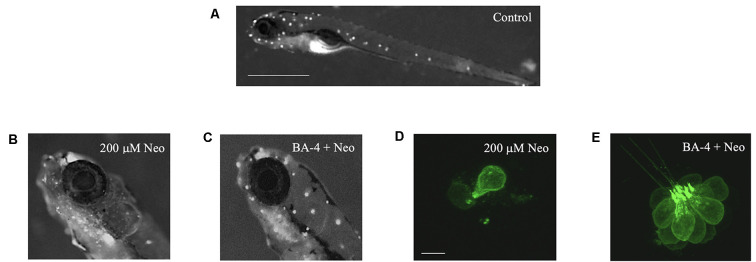
Berbamine analogs confer protection from neomycin. Analog BA-4 is shown here as an example.** (A)** Zebrafish labeled with the mitochondrial dye DASPEI to visualize neuromasts in the lateral line (white dots). Scale bar = 1 mm. **(B)** Zebrafish labeled with DASPEI after 200 μM neomycin resulting in reduced fluorescence. **(C)** Zebrafish labeled with DASPEI after pre-co treatment with analog BA-4 and 200 μM neomycin. **(D,E)** Representative IO3 neuromasts from *Tg(Brn3c:GFP)* zebrafish. **(D)** Zebrafish exposed to 200 μM neomycin exposure, resulting in hair cell loss. Scale bar = 10 μm. **(E)** Zebrafish exposed to a pre-co treatment of analog BA-4 and 200 μM neomycin.

**Figure 3:**
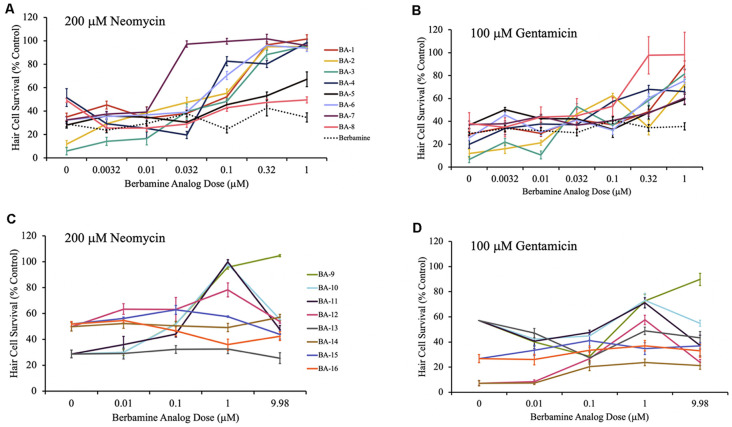
Berbamine analogs confer protection from aminoglycosides. **(A,B)** Dose-response curves for berbamine analog polymers and berbamine (parent compound) against 200 μM neomycin and 100 μM gentamicin. **(C,D)** Dose-response curves for the remainder of the berbamine analog polymers, and the berbamine analog monomers (BA-13 to BA-16) against 200 μM neomycin and 100 μM gentamicin. Zebrafish were pretreated for 1 h with each berbamine analog at variable doses followed by co-treatment with analog and aminoglycoside. Data are normalized to controls. Twelve analogs protected hair cells from neomycin damage, while 11 protected from gentamicin. Ten of the berbamine analogs were protective against both neomycin and gentamicin in a dose-dependent manner. Hair cells were assessed *via* DASPEI. Data are presented normalized to vehicle controls. Data were analyzed by one-way ANOVA, *N* = 4–12, except for the gentamicin dose-response curves for BA-9, BA-10, BA-11, and BA-13, where *N* = 2 for the gentamicin-only groups. We are still confident in the data for these groups, based on the additional gentamicin data shown here and the data in Supplementary Figure S1. Bars are ± SEM. Refer to Table 1 for statistics.

We examined these 16 different berbamine analogs and found that all 12 acylated or alkylated analogs (BA-1 to BA-12) protected hair cells from at least one of the aminoglycosides (Figures 2, 3) and were more robust protectants than berbamine. Twelve total analogs significantly protected against acute neomycin damage, while 13 conferred protection from chronic gentamicin. The berbamine monomer benzyltetrahydroisoquinolines conferred little to no protection; BA-14 was significantly protective from gentamicin, and BA-15 was significantly protective from neomycin, but in both cases, protection was modest compared to the other analogs. We observed some toxicity for some analogs for the highest concentration tested (9.98 μM) but all protective concentrations were orders of magnitude lower than the ototoxic concentration.

Given our long-term goal of clinical translation, we were especially interested in the analogs with both high efficacy and high potency. As shown in Figure 3, for all 11 acylated or alkylated analogs that attenuated neomycin damage, the OPC (optimal protective concentration) was between 0.032 μM and 1 μM with an ED_50_ range of 0.02 μM–0.24 μM (Figures 3A,C). BA-7 (purple line) was the most potent analog with an OPC of 0.032 μM and an ED_50_ of 0.02 μM. All 12 acylated or alkylated analogs reduced gentamicin toxicity with OPCs varying between 0.32 μM and 9.98 μM and an ED_50_ range of 0.03 μM–1.83 μM (Figures 3B,D). Compound BA-8 (pink line) was an exceptionally robust protectant, with an OPC of 0.32 μM against gentamicin, yet no protection from neomycin. Overall, the phenyl ring appears to be important, but BA-9, which is an alkylated version of berbamine (and an extension of the alkyl group of isotetrandrine) is also robustly protective, so the phenyl group may not be needed. The statistical analyses for all neomycin and gentamicin dose-response curves can be found in Table 1. Collectively, these data suggest that acylated and alkylated berbamine analogs are important for hair cell protection due to the bisbenzylisoquinoline ring system and the hydrophobic functional groups.

**Table 1:** Statistics for berbamine analog dose-response curves for 200 μM neomycin and 100 μM gentamicin for the data shown in Figure 3.

Compound Name	Neomycin: ANOVA	Neomycin: *P*-value by concentration (μM)	Gentamicin: ANOVA	Gentamicin: *P*-value by concentration (μM)
**BA-1**	*F*_(6,69)_ = 94.79 *P* < 0.0001	0.1; ** 0.32; **** 1; ****	*F*_(6,70)_ = 48.91 *P* < 0.0001	0.032; * 0.32; **** 1; ****
**BA-2**	*F*_(6,66)_ = 72.23 *P* < 0.0001	0.0032; ** 0.01; **** 0.032; **** 0.1; **** 0.32; **** 1; ****	*F*_(8,89)_ = 50.76 *P* < 0.0001	0.032; **** 0.1; **** 0.32; *** 1; **** 3.16; **** 9.98; ****
**BA-3**	*F*_(6,70)_ = 70.76 *P* < 0.0001	0.032; *** 0.1; **** 0.32; **** 1; ****	*F*_(8,63)_ = 61.08 *P* < 0.0001	0.0032; * 0.032; **** 0.1; **** 0.32; **** 1; **** 3.16; **** 9.98; ****
**BA-4**	*F*_(6,70)_ = 51.23 *P* < 0.0001	0.0032; ** 0.01; ** 0.032**** 0.1; **** 0.32; *** 1; ****	*F*_(6,60)_ = 19.36 *P* < 0.0001	0.01* 0.032; * 0.1; **** 0.32; **** 1; ****
**BA-5**	*F*_(6,68)_ = 15.29 *P* < 0.0001	0.1; ** 0.32; **** 1; ****	*F*_(6,42)_ = 4.34 *P* = 0.017	0.0032; * 1; ***
**BA-6**	*F*_(6,70)_ = 94.12 *P* < 0.0001	0.1; **** 0.32; **** 1; ****	*F*_(6,76)_ = 41.35 *P* < 0.0001	0.0032; **** 0.032; *** 0.32; **** 1; ****
**BA-7**	*F*_(6,53)_ = 113.8 *P* < 0.0001	0.032; **** 0.1; **** 0.32; **** 1; ****	*F*_(8,77)_ = 10.83 *P* < 0.0001	1; *** 3.16; ****
BA-8	*F*_(6,72)_ = 19.58 *P* < 0.0001	0.0032; **** 0.01; **** 0.032; ****	*F*_(8,59)_ = 19.85 *P* < 0.0001	0.32; **** 1; **** 3.16; ****
**BA-9**	*F*_(4,40)_ = 332.3 *P* < 0.0001	0.1; **** 1; **** 9.98; ****	*F*_(4,38)_ = 32.05 *P* < 0.0001	0.1; * 9.98; *
**BA-10**	*F*_(4,39)_ = 111.5 *P* < 0.0001	0.1; **** 1; **** 9.98; ****	*F*_(4,33)_ = 9.09 *P* < 0.0001	ns
**BA-11**	*F*_(4,26)_ = 87.80 *P* < 0.0001	0.1; ** 1; **** 9.98; **	*F*_(4,23)_ = 7.09 *P* = 0.0007	ns
**BA-12**	*F*_(4,37)_ = 5.90 *P* = 0.0009	1; ***	*F*_(4,52)_ = 67.35 *P* < 0.0001	0.1; **** 1;**** 9.98****
BA-13	*F*_(4,32)_ = 0.60	Ns	*F*_(4,22)_ = 5.18 *P* = 0.004	0.1; **
BA-14	*F*_(4,44)_ = 0.468	Ns	*F*_(4,48)_ = 10.54 *P* < 0.0001	0.1; *** 1; **** 9.98; ***
BA-15	*F*_(4,48)_ = 8.03 *P* < 0.0001	0.1; **	*F*_(5,34)_ = 1.80 *P* = 0.139	ns
BA-16	*F*_(4,40)_ = 5.99 *P* = 0.0007	1; **	*F*_(5,34)_ = 1.29 *P* = 0.289	ns
Berbamine	*F*_(6,60)_ = 4.02 *P* = 0.0019	0.32*	*F*_(6,66)_ = 2.14 *P* = 0.06	ns

Compound names in *bold* indicate protection from both neomycin and gentamicin, while compound names in black (not bold) protect from neomycin *or* gentamicin, and compound names in gray indicate non-protective compounds. Data were analyzed by one-way ANOVA (columns 2 and 4) followed by Bonferroni-corrected *t*-tests for multiple comparisons (columns 3 and 5). In columns 3 and 5, numbers in black indicate significant increases in hair cell survival (compared to aminoglycoside-only controls), while numbers in gray indicate significant reductions in hair cell survival relative to aminoglycoside-only groups. ****p < 0.0001, ***p < 0.001, **p < 0.01, *p < 0.05. ns, not significant.

### Hair Cells Are Viable and Functional After Prolonged Analog Exposure

**Figure 4:**
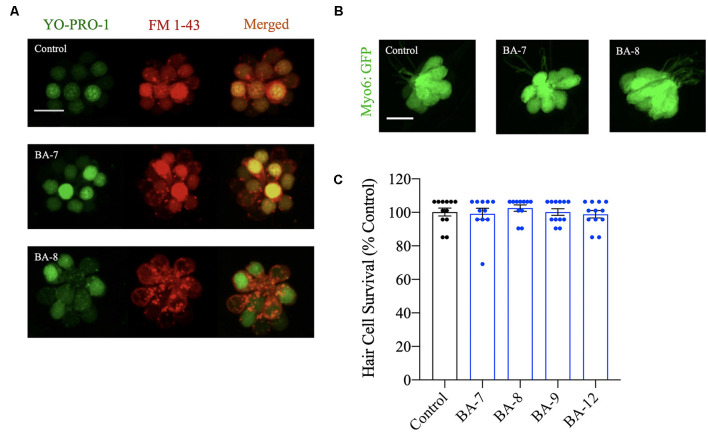
Hair cells are viable after analog exposure. **(A)** Zebrafish were pretreated with the nuclear marker YO-PRO-1 for 1 h followed by treatment with the optimally protective concentration of each analog for 24 h. Zebrafish were then rinsed in embryo medium (EM) and treated with FM 1–43FX to assess mechanoelectrical transduction (MET) channel function after prolonged analog exposure and removal. Analog treatment did not alter FM 1–43FX or YO-PRO-1-labeling. Scale bar = 10 μm. **(B)** Examples of *Tg(myo6b:EGFP)* neuromasts treated with the optimally protective concentration of each analog for 24 h before hair cell assessment. In each case, neuromast morphology appeared normal. Scale bar = 10 μm. **(C)** Zebrafish were exposed to the optimal protective concentration (OPC) of berbamine analog for 24 h before hair cell assessment with DASPEI. None of the analogs impact hair cell survival. Data are normalized to controls and analyzed by one-way ANOVA, *F*_(4,48)_ = 0.6564, *p* = 0.6252. *N* = 11–12, bars are ± SEM.

Next, we wanted to assess whether our analogs were compromising the long-term viability of the hair cells or the functionality of the MET channel after prolonged analog exposure and removal. After a 24-h incubation in each analog at the OPC, we assessed hair cell viability using the vital dye YO-PRO-1 and found no qualitative reduction in fluorescence intensity (Figure 4A). FM 1–43FX was used to assess MET channel function since this dye is known to enter hair cells *via* the MET channel (Gale et al., 2001; Alharazneh et al., 2011). Of the analogs tested that showed protection from neomycin and/or gentamicin, none of them appeared to reduce FM 1–43FX entry, suggesting that MET channel integrity is preserved after analog removal (Figure 4A). To further complement this experiment, berbamine analogs were individually administered for 24 h to *myo6b:EGFP* fish to observe hair cell morphology or to AB larval zebrafish for hair cell survival assessment with DASPEI labeling (Figures 4B,C). We did not observe changes to hair cell morphology or reductions in DASPEI scores. Overall, our viability and functional assays indicate that at their OPC, our analogs do not cause hair cell toxicity.

**Figure 5:**
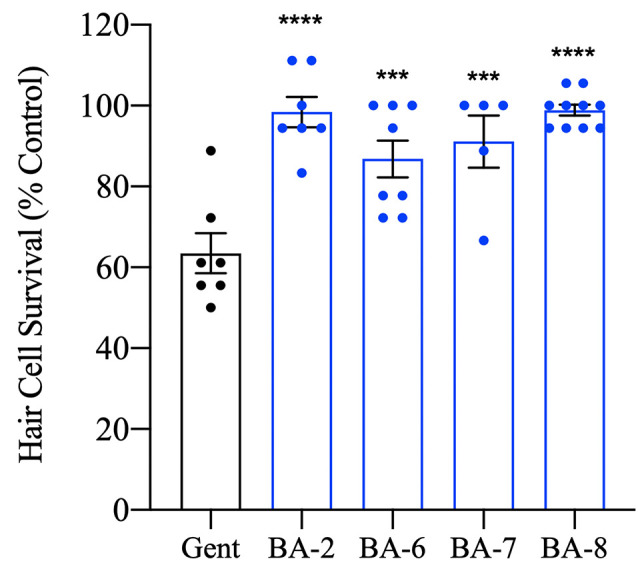
Hair cell protection persists after 24 h. Zebrafish were pretreated with the OPC of analog for 1 h followed by 200 μM gentamicin treatment for 30 min. Fish were allowed to recover for 24 h before the assessment. All analogs tested significantly prevented hair cell death after 24 h suggesting true protection rather than a delay in cell death onset. Hair cells were assessed *via* DASPEI. Data are presented normalized to vehicle controls. Data were analyzed by one-way ANOVA, ****p < 0.0001, ***p < 0.001, *F*_(4,32)_ = 12.98, *N* = 5–10, bars are ± SEM.

Given that our protective analog concentrations are not inherently ototoxic, we next wanted to test whether berbamine analogs were truly otoprotective or if they only delayed hair cell death. We therefore pre-treated larvae with the OPC of analog for one hour before a 30-min administration of gentamicin, then assessed hair cells 24 h later. This time course was selected because after an acute gentamicin exposure hair cell damage continues even after drug removal (Owens et al., 2009). All of the analogs tested (BA-2, BA-6, BA-7, and BA-8) showed over 90% hair cell survival 24 h post-gentamicin exposure compared to untreated controls (Figure 5). When we compared the protection of our analogs in chronic (6 h) vs. acute (30 min + 24 h) gentamicin treatment, we saw that BA-6 and BA-7 exhibited 10–20% more protection in the acute gentamicin exposure assay (compare Figures 3B, 5). These data demonstrate that berbamine analogs robustly protect hair cells from aminoglycoside damage after both acute and chronic aminoglycoside treatment.

### A Subset of Berbamine Analogs Reduce GTTR Loading

**Figure 6:**
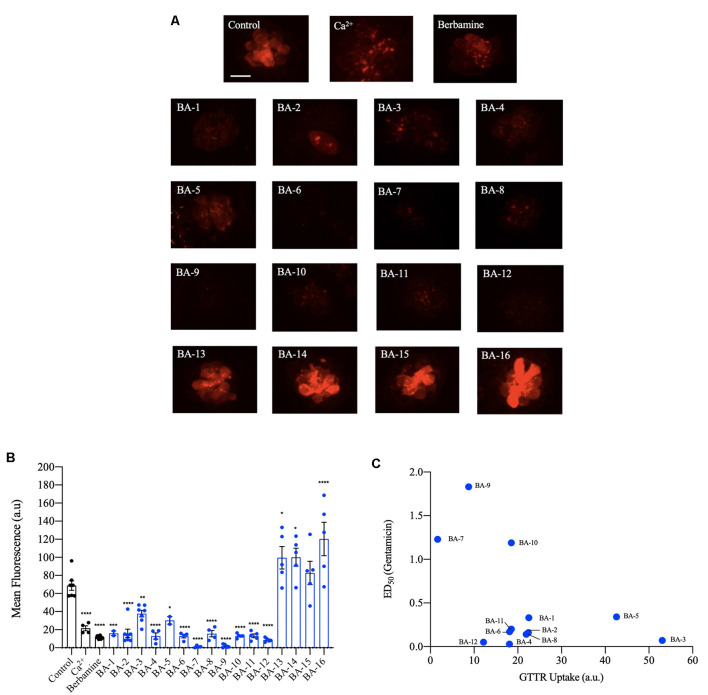
Berbamine analogs reduce aminoglycoside uptake by hair cells. **(A)** Twelve of the analogs significantly reduced uptake of gentamicin conjugated with Texas Red (GTTR), as did berbamine. Zebrafish were pretreated with the optimally protective analog concentration (or with 25 μM berbamine, Kruger et al., 2016) for 1 h followed by a co-treatment with 50 μM GTTR for 18 min. Dimethylsulfoxide (DMSO) and high calcium are negative and positive controls, respectively. **(B)** Fluorescence intensity quantification using the OPC of analog. The intensity was measured in arbitrary units and the data were normalized by subtracting the background fluorescence. Data were analyzed *via* one-way ANOVA, *F*_(17,101)_ = 38.71, ****p < 0.0001, ***p < 0.001, **p < 0.01, *p < 0.05, asterisks denote significantly different from vehicle controls, *N* = 4–8 for most compounds, except for *N* = 2 for compounds BA-1 and BA-5 and *N* = 15 for berbamine, bars are ± SEM. **(C)** Linear regression was conducted for analogs that protect hair cells from gentamicin to identify a relationship between analog potency and uptake (*y* = −0.0203x + 0.9162). The Pearson correlation coefficient of 0.2268 denotes no significant relationship. Berbamine data are replotted from Kruger et al. (2016).

Next, we investigated if our analogs protect hair cells by blocking aminoglycoside entry. Previous research shows that aminoglycosides predominantly enter hair cells *via* the MET channel. Furthermore, quinoline-ringed structures have been shown to protect hair cells by reducing MET channel-mediated aminoglycoside loading (Ou et al., 2012; Kirkwood et al., 2017). Since berbamine is a quinoline ring structure we predicted its analogs could also protect by reducing aminoglycoside uptake *via* the MET channel. We found that all 12 of the acylated or alkylated berbamine analogs (BA-1 to BA-12) attenuated GTTR uptake by 50% or more relative to negative controls (Figures 6A,B). The berbamine monomers (BA-13 to BA-16) did not attenuate uptake compared to negative controls. Calcium, which reduces the open probability of the MET channel, was used as a positive control. While calcium reduced GTTR by 70%, 10 of our analogs were more effective uptake blockers, as was the parent compound berbamine (Figure 6B, Kruger et al., 2016). BA-7 was the most effective uptake blocker with over 95% attenuation of GTTR fluorescence (Figure 6B). We then conducted a linear regression to determine how strongly hair cell protection was correlated with GTTR uptake (Figure 6C). Given a correlation coefficient of 0.23, there is no relationship, suggesting that protection may not be solely reliant on aminoglycoside uptake block. Overall our GTTR uptake analysis indicates that a subset of analogs block GTTR loading into the hair cells but the magnitude of uptake block is not directly correlated with hair cell protection.

### Berbamine Analogs Differentially Affect Mechanoelectrical Transduction Channel Activity

**Figure 7:**
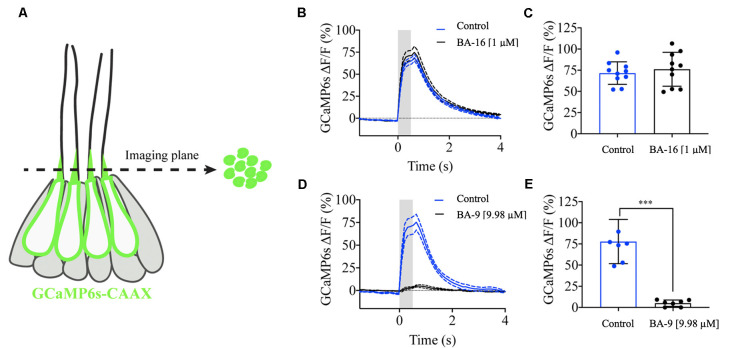
Berbamine analogs differentially reduce mechanotransduction channel activity. Mechanosensitive-calcium signals from hair bundles of posterior lateral line neuromasts. **(A)** Side view of a neuromast expressing hair cell-specific membrane-localized GCaMP6s. Left: an imaging plane through the apical hair bundles can be used to measure mechanosensitive-calcium signals during fluid-jet stimulation. Right: a top-down view of the imaging plane showing the hair bundles expressing GCaMP6s. **(B)** Traces comparing the average GCaMP6s signals before and after application of 1 μM BA-16, *N* = 10 neuromasts. **(C)** Dot plots quantifying the peak GCaMP6s response per neuromast. BA-16 does not alter evoked calcium activity in hair cells. **(D)** Traces comparing the average GCaMP6s signals before and after application of 1 μM BA-9, *N* = 7 neuromasts. **(E)** Dot plots quantifying the peak GCaMP6s response per neuromast. BA-9 reduces MET-evoked calcium activity compared to baseline levels. Each dot in **(C,E)** averages the response of all hair bundles in each neuromast. A paired *t*-test was used in **(C,E)**, ***p < 0.001. Error bars in **(C,E)** and dashed lines on either side of the solid line in **(B,D)** represent ± SEM.

Our results indicate that several of our otoprotective berbamine analogs attenuate aminoglycoside toxicity by blocking GTTR uptake through MET channels. To confirm that protective analogs impair mechanotransduction, we mechanically stimulated lateral line hair cells and measured GCaMP6s-evoked calcium signals in hair bundles (Lukasz and Kindt, 2018). This previously established method allows for sensitive and quantitative measurements of mechanotransduction in lateral line hair bundles (Figure 7A). Therefore, these GCaMP6s signals can provide a quantitative readout of mechanosensitive-calcium responses in the presence and absence of berbamine analogs. For our analysis, we tested two representative analogs, one protective analog (BA-9, a potent GTTR blocker) and one analog that was not protective (BA-16, which does not block GTTR uptake). We first measured evoked GCaMP6s signals in hair bundles during fluid-jet stimulation before analog treatment (control, blue line, Figures 7B–E). After the application of the berbamine monomer BA-16, there was no difference in the magnitude of the GCaMP6s signals in hair bundles as compared to the pretreatment condition, indicating that BA-16 does not impair mechanotransduction (Figures 7D,E). By contrast, the application of the berbamine derivative BA-9 reduced the GCaMP6s signals by ~95%, indicating significant inhibition of mechanotransduction (Figures 7B,C). Our calcium imaging experiments complement the GTTR uptake data and suggest that when berbamine analogs block GTTR uptake, their otoprotective effects are due to the MET channel block, thereby reducing aminoglycoside entry into hair cells.

### Berbamine Analogs May Also Protect *via* Uptake-Independent Mechanisms

**Figure 8:**
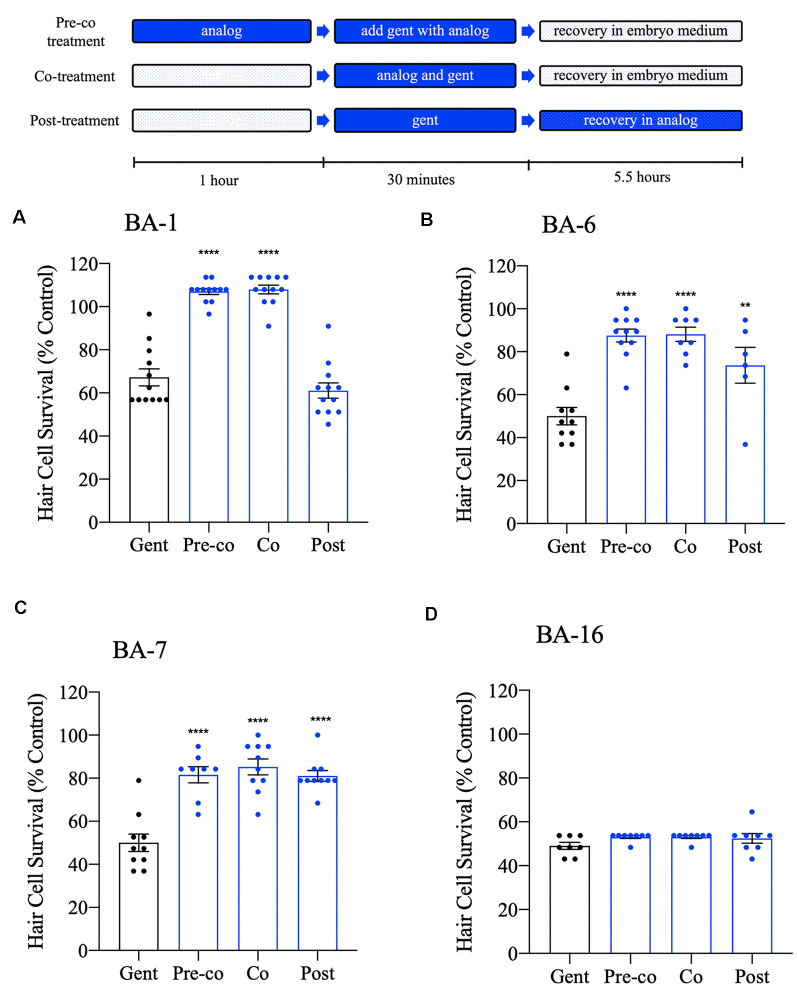
Differential patterns of analog protection from gentamicin. Zebrafish were either pre-treated with the OPC of analog for 1 h, then co-treated with analog and gentamicin for 30 min (“pre-co” condition), co-treated with analog and gentamicin without a pretreatment period (“co” condition) or post-treated with analog for 5.5 h following gentamicin removal (“post” condition). All treatments used 200 μM gentamicin. Examples are shown for analogs that represent each pattern of protection. **(A)** Analog BA-1 was only protective when administered in the “pre-co” or “co” conditions suggesting that protection occurs *via* aminoglycoside uptake block. **(B,C)** Analogs BA-6 and BA-7 significantly protected hair cells in all three conditions, suggesting an intracellular mechanism of protection in addition to aminoglycoside uptake block. **(D)** Analog BA-16 is not protective of any treatment paradigm. Hair cells were assessed *via* DASPEI. Data are presented normalized to untreated controls, which are not shown but represent 100% hair cell survival. Data were analyzed *via* one-way ANOVA, ****p < 0.0001, **p < 0.01 (as compared to gentamicin-only controls), *N* = 7–12, bars are + SEM. Refer to Supplementary Table S1 for statistics.

We have shown that many of our berbamine analogs can confer protection when co-administered with gentamicin. Given that 12 of our berbamine analogs are likely to be otoprotective due to reduced GTTR uptake, we hypothesized that no protection would be conferred if we administered the analogs after gentamicin removal. To test this hypothesis, we used an acute 30-min gentamicin treatment time course. We compared three gentamicin treatment paradigms: (1) 1-h pre-treatment with the analog, followed by co-treatment with analog and gentamicin (pre-co); (2) co-treatment with the analog-only during gentamicin treatment (no pre-treatment; co) and (3) analog post-treatment for 5.5 h *after* gentamicin washout (post). All experiments used the OPC of each analog (Figure 8).

Consistent with our hypothesis, eight of our analogs (BA-1, BA-2, BA-4, BA-8, BA-9, BA-10, BA-11, and BA-12) only protected hair cells when the analog was administered before and during (pre-co) or concurrent with (co) aminoglycoside administration (Figures 8A–C, Supplementary Figure S1, Supplementary Table S1). One analog, BA-5, which was previously not protective when co-administered with gentamicin in the chronic assay, was protective when administered before and during gentamicin exposure (pre-co). This suggests that protection occurs *via* aminoglycoside uptake block in an acute gentamicin administration but that protection diminishes in a longer treatment paradigm. Surprisingly, BA-3, BA-6, and BA-7 showed protection in all three treatment paradigms. Analogs that show protection even when administered after aminoglycoside removal suggest that hair cell protection may also occur by an uptake-independent mechanism such as interaction with intracellular pathways.

### Berbamine Analogs Enter Hair Cells

**Figure 9:**
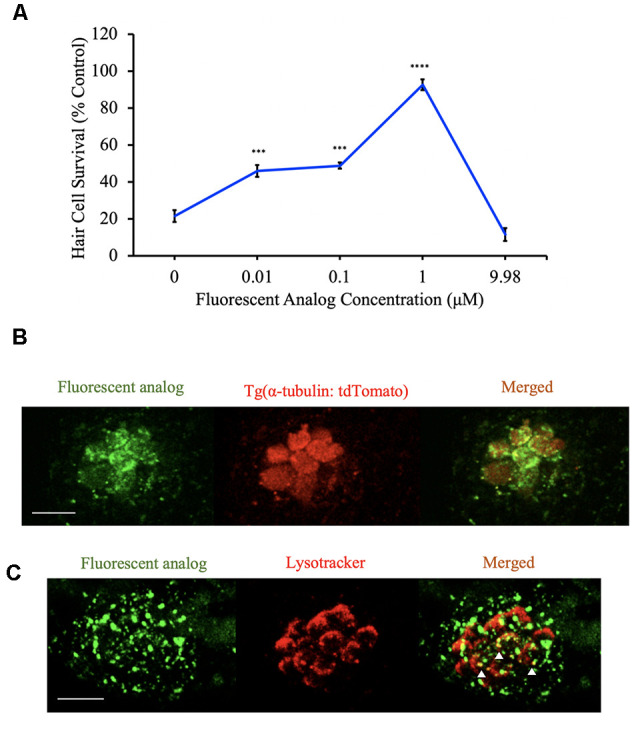
Berbamine analogs enter hair cells. **(A)** The dose-response curve for the fluorescent analog (BA-17) demonstrates significant protection from 200 μM neomycin. Hair cells were assessed *via* DASPEI. Data are normalized to controls. One-way ANOVA, *F*_(4,49)_ = 50.22, *p* < 0.0001. *N* = 10–12, ****p < 0.0001, ***p < 0.001, bars are ± SEM. **(B)** *Tg(α-tubulin: tdTomato)* zebrafish were pretreated with BA-17 for 1 h before imaging on a confocal microscope. Fluorescent analog (green) is present in hair cells (red). Scale bar = 10 μm. **(C)** AB zebrafish were pretreated with BA-17 for 1 h followed by a 3-min exposure to 50 nM LysoTracker before confocal imaging. Fluorescent analog (green) does overlap with lysosomes (red) in some areas (white arrowheads). Scale bars in **(B,C)** = 10 μm.

Given that a subset of analogs protects hair cells by both uptake-dependent and uptake-independent mechanisms, we next wanted to know if the analogs enter hair cells. For these experiments, we used the approach outlined in Figure 1 to synthesize BA-17, a berbamine analog with a fluorescent 7-(diethylamino)coumarin tag. To assess for protection by this fluorescent analog, zebrafish were exposed to varying concentrations of analog before and concurrently with aminoglycoside exposure. We found that BA-17 was protective in a dose-dependent manner against 200 μM neomycin and toxic at a concentration of 9.98 μM, consistent with other protective analogs (Figure 9A). To determine whether our analogs enter hair cells, we pre-treated *Tg(α-tubulin: tdTomato)* zebrafish with the OPC of BA-17 for one hour before confocal imaging. BA-17 was visible in hair cells as well as some punctae in surrounding neuromasts, suggesting the analog can enter multiple cell types (Figure 9B). To begin to understand cellular localization of berbamine analogs, we asked if our analogs enter lysosomes. For these experiments, we pre-treated AB zebrafish for 1 h with BA-17 followed by incubation in LysoTracker and found that some BA-17 punctae colocalize with the lysosomal marker (Figure 9C). These data demonstrate that berbamine analogs can enter neuromasts cells and localize in subcellular compartments, including lysosomes.

## Discussion

Our former work demonstrated that berbamine, a natural plant alkaloid, robustly protected zebrafish hair cells from aminoglycoside-induced damage (Kruger et al., 2016). In a separate study, Kirkwood et al. (2017) further confirmed berbamine’s otoprotective properties in the zebrafish lateral line and in mouse cochlear explants. The present study set out to develop berbamine analogs that are more potent and efficacious than the parent compound and to investigate the extent of their protection against aminoglycoside-induced hair cell damage. The medicinal chemistry campaign strategy focused on functionalizing the phenol group to synthesize analogs as an initial probe of the structure-activity relationship. Leveraging available chemistry in this manner is a robust strategy employed in the early development of natural products. Like many natural products, the berbamine scaffold is somewhat complex making *de novo* synthesis far more time consuming and expensive than substitution at the available phenol. E6 berbamine and isotetrandrine, which also confer protection from aminoglycosides (Kruger et al., 2016), are both substituted at the phenol suggesting further exploration at that position would likely produce active compounds. Moreover, since MET inhibition was the likely target of activity, increasing analog size and hydrophobicity by substitution at the phenol should make analogs better-MET blockers.

**Figure 10:**
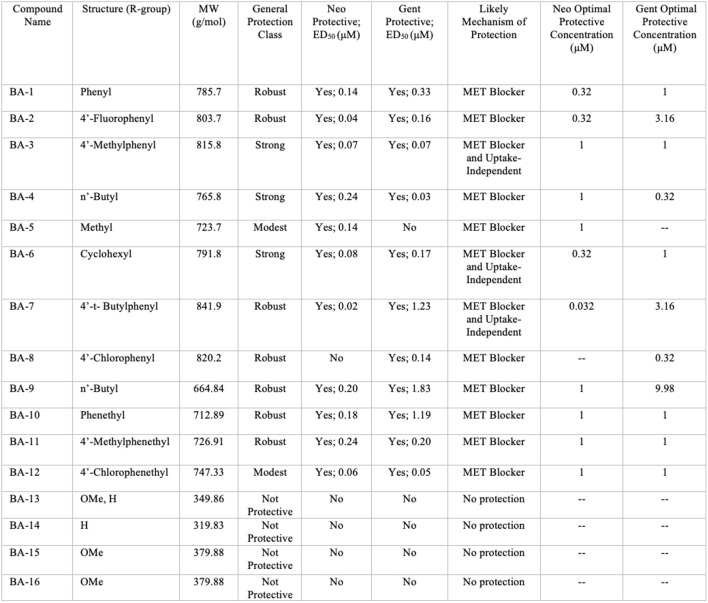
Summary of berbamine analog protection and compound characteristics. Compound name, structure, molecular weight, protection class, likely mechanism of protection, ED_50_, and OPCs against neomycin and gentamicin for all 16 berbamine analogs. The general protection classes were defined based on thresholds of protection levels by percent. Robust = 60% increase in hair cell protection compared to aminoglycoside group; Strong = over 40% increase in hair cell protection compared to aminoglycoside group; Modest = over 30% increase in hair cell protection compared to aminoglycoside group; No protection = less than 30% of hair cell protection compared to aminoglycoside group. The likely mechanism was determined by the GTTR experiments in Figure 6 and the time course experiments shown in Figure 8 and Supplementary Figure S1.

Overall, we show that many of our berbamine analogs effectively protect hair cells from aminoglycoside toxicity, demonstrating more robust protection than berbamine. Details of all 16 non-fluorescent analogs are shown in Figure 10, where we classify analogs based on the relative degree of protection. Of the 16 analogs, nine demonstrated at least 40% protection against both neomycin and gentamicin-induced hair cell death (“robust” or “strong” protection, see Figure 10). Two analogs protected against neomycin (>30% protection) but conferred little protection from gentamicin (BA-5, BA-12) and one only conferred protection from gentamicin but not neomycin (BA-8). Twelve analogs reduced GTTR and thus likely confer protection by blocking aminoglycoside entry through the MET channel, which is the primary site of aminoglycoside entry into hair cells (Marcotti et al., 2005; Alharazneh et al., 2011). BA-9, a potent uptake blocker and protective compound against both neomycin and gentamicin, reduced MET-evoked calcium activity to baseline levels, further supporting our hypothesis that protection is largely due to MET channel block. The reduction of MET-evoked calcium activity was not observed with BA-16, a non-protective compound. Our results are consistent with Kirkwood et al. (2017), who demonstrated that berbamine is a permeant MET channel blocker in mouse cochlear hair cells.

Attenuating aminoglycoside uptake into hair cells has gained traction as a feasible otoprotective strategy (Ou et al., 2012; O’Sullivan et al., 2017; Kitcher et al., 2019). d-Tubocurarine and phenoxybenzamine have previously been shown to protect hair cells by reducing aminoglycoside entry (Kirkwood et al., 2017; Majumder et al., 2017). d-Tubocurarine is a structural analog of berbamine with a dimethyl quaternary ammonium ion in one of the tetrahydroisoquinoline rings and with one of the other methoxy groups replaced with a second phenol (Supplementary Figure S2). Ionic compounds such as d-tubocurarine might be expected to block the MET channel since similar effects were observed with analogs of other scaffolds (Supplementary Figure S2). ORC-13661 is being developed as an otoprotectant and also protects both zebrafish and rodent hair cells from aminoglycosides, likely by blocking aminoglycoside uptake (Chowdhury et al., 2018). Quaternary analogs of ORC-13661 were much more active than the non-quaternary versions. ORC-13661 as well as our berbamine analogs contain lipophilic groups that appear to improve protective activity. Protection is likely due to the hydrophobic nature of the compounds and their ability to diffuse through plasma membranes. This attribute makes these compounds better candidates for future drug development since they could potentially cross the blood-brain barrier and/or the blood labyrinth barrier (Abbott et al., 2006; Koo et al., 2015; Nyberg et al., 2019).

The majority of our protective analogs were not only lipophilic but also contained an aryl group. Absence of the aryl moiety reduced our analogs’ overall protection from aminoglycosides, especially from gentamicin, as we found for compound BA-5. Loss of an aryl substituent and its impact on protective activity from aminoglycosides in zebrafish was also observed in Chowdhury et al. (2018). In that study, the original screening hit contained a *p*-chlorophenyl group and replacement led to a loss of activity. In our study, analog BA-8 also contained a chlorophenyl substituent but it did not seem to be important for neomycin protection, in contrast to the Chowdhury et al. (2018) study. However, the chlorophenyl substituent was important for robust gentamicin protection. Likely, differences seen in neomycin protection for the p-chlorophenyl compounds (ORC-13661 and BA-8) may have more to do with how the substituent interacts in combination with its compound scaffold to permeate the MET channel rather than channel blocking activity being solely reliant on the moiety.

Nevertheless, aryl groups are present in other protective compounds such as tacrine, berbamine, carvedilol, ORC-13661, and phenoxybenzamine, suggesting the importance of the aryl moiety for otoprotection (Ou et al., 2012; Kruger et al., 2016; Chowdhury et al., 2018; O’Reilly et al., 2019). It is likely that the phenyl group, among other aryl groups, may be necessary for protection due to their unusual stability and ability to delocalize charges within their structure by resonance. This would allow for temporary charges to reside within the structure which may form favorable electrostatic interactions with its target and could explain why quaternary versions of protective drugs such as ORC-13661 are more active. This is further supported by evidence from Chowdhury et al. (2018) and O’Reilly et al. (2019). Both studies found that aminoglycoside otoprotection required a basic center with a positive charge to engage in electrostatic interactions, such as basic nitrogen or a quaternary ammonium salt. An electronegative atom could also yield a temporary dipole and thus a transient charge, which may explain why analogs such as BA-2 and BA-8 were robust MET blockers. However, aromaticity is not the only feature that contributes to otoprotection. The majority of our aromatic compounds were not protective, such as BA-13 through BA-16. These compounds were half the weight of the other analogs tested. Additionally, analogs BA-13 through BA-16 have a carbon center, similar to some non-protective carvedilol derivatives that substituted the center nitrogen with carbon or nonbasic nitrogen (O’Reilly et al., 2019).

BA-8 robustly protects hair cells from gentamicin damage yet confers no protection from neomycin. Also, the majority of our protective analogs show differing degrees of protection from neomycin vs. gentamicin, supporting the literature that neomycin and gentamicin activate partially distinct pathways of hair cell damage (Owens et al., 2009; Coffin et al., 2013a,b; Wiedenhoft et al., 2017). BA-5 was only protective when administered before and during gentamicin exposure (pre-co), suggesting protection occurs *via* transient aminoglycoside uptake block during acute gentamicin administration. In the chronic gentamicin assay, BA-5 was not protective against gentamicin. This could mean that BA-5 may disassociate from the MET channel in a longer treatment paradigm thus rendering it non-protective. These data are consistent with prior studies showing that acute neomycin and acute gentamicin activate similar damage mechanisms, while chronic gentamicin also operates *via* additional pathway(s) (Coffin et al., 2009, 2013a,b; Owens et al., 2009). Additionally, there is more recent evidence that aminoglycosides enter hair cells *via* both endocytic and non-endocytic pathways, resulting in drug delivery from the extracellular space to lysosomes, and that intracellular trafficking differs between aminoglycosides (Hailey et al., 2017). Some of our analogs likely target cellular pathways that are unique to neomycin vs. gentamicin, thus accounting for the observed differences in protection.

We observed that the fluorescently modified analog BA-17 does enter hair cells but whether the entry is necessary for protection remains to be determined. An interesting observation from this experiment was the punctate/granular labeling of BA-17 in hair cells, which may co-localize with lysosomes (Figure 9C), supporting findings from Hailey et al. (2017) that both aminoglycosides that enter hair cells ultimately end up in lysosomes in zebrafish. Future experiments will further examine the analogs’ mechanism of protection by observing how long the fluorescently tagged analog stays in hair cells and how that time scale compares to the protection that it exhibits. We also found that there is no correlation between the degree of uptake block and the magnitude of hair cell protection. Interestingly, some of the analogs that weren’t protective (BA-13, BA-16) seemed to enhance GTTR entry. This information could be potentially useful as it suggests that certain moieties may exacerbate toxicity and should be avoided in future drug optimization. Additionally, our analog washout experiments suggest that our analogs disassociate from the channel and do not alter MET channel function after the analog is removed, suggesting that hair cell viability is preserved.

While the MET channel is the primary site of aminoglycoside entry into hair cells, several other ion channels are also candidates for aminoglycoside entry. Transient receptor potential (TRP) channels such as TRPV1, TRPV4, and TRPA1 are particularly interesting because they have pore diameters large enough to allow passage of bulky aminoglycoside molecules (Myrdal and Steyger, 2005; Karasawa et al., 2008; Stepanyan et al., 2011). Kenyon et al. (2017) discovered a select group of aromatic compounds that protect against aminoglycoside-induced hearing loss and are also known to have biological activity for potassium channels, NMDA and AMPA receptors, and other channels. Of the 13 protective analogs identified in their study, only six were MET channel blockers, yet additional compounds reduced fluorescent drug entry, implying that the other compounds may exert their protective effects through other receptors or channels (Kenyon et al., 2017). Bisbenzylisoquinoline compounds, in addition to inhibiting prostaglandins and leukotrienes (Teh et al., 1990) are also proficient vasodilators and modulators of voltage-gated calcium channels (Medeiros et al., 2011; Alaoui et al., 2015), leading to the hypothesis that our analogs may reduce aminoglycoside entry through alternate channels in tandem with the MET channel, which would explain the varying degrees of GTTR fluorescence attenuation shown by our analogs. This would also explain why some of our multimodal protective analogs such as BA-3 and BA-6 are not the strongest of uptake blockers, unlike analogs such as BA-9 and BA-12, which only protect by attenuating aminoglycoside entry. Future studies will further characterize the binding targets of our berbamine analogs, which can also provide insight into the mechanisms of aminoglycoside-induced hair cell death.

Based on our findings that the majority of our analogs protect by blocking aminoglycoside uptake, we expected to only observe protection when the berbamine analog was administered either before or at the same time as the antibiotic. We were surprised that three analogs (BA-3, BA-6, and BA-7) exhibited protection when delivered after aminoglycoside exposure but before hair cell death, suggesting both uptake-dependent and uptake-independent protective mechanisms. Interestingly, all three analogs were alkylated with bulky lipophilic groups such as alkyl-substituted phenyl groups and cyclohexyl analog. Analogs with halide substitution or small straight-chain alkyl substitution did not have the same profile. Berbamine has been used as an anti-inflammatory in traditional Chinese medicine and has gained traction as an anti-cancer agent (Du et al., 2018). Previous studies have shown that berbamine and its analogs can inhibit tumor growth by modulating nuclear factor kappa-light-chain-enhancer of activated B cells (NF-κB) signaling (Liang et al., 2016; Jia et al., 2017). A recent study showed that berbamine has anti-inflammatory activity that robustly inhibits the release of chemokines and cytokines including TNF-α, IL-1β, IL-6 (Jia et al., 2017). Berbamine also suppressed the phosphorylation of p65, IκB, c-Jun N-terminal kinase (JNK) pathways, and extracellular receptor kinase (ERK)1/2 pathways (Jia et al., 2017), all of which are implicated in aminoglycoside-induced hair cell loss (Huth et al., 2011). Possibly the berbamine analogs that confer uptake-independent protection may reduce pro-inflammatory cytokines or modulate pathways such as NF-κB, JNK, or ERK1/2. Future work will examine the berbamine modulation of intracellular pathways in aminoglycoside-damaged hair cells.

In our prior work, 25 μM berbamine was the optimal concentration that prevented aminoglycoside toxicity and reduced GTTR loading (Kruger et al., 2016 and Figure 6A). One advantage of our analogs compared to berbamine is that the analogs are protective at a much lower concentration than the parent compound (over 100-fold more potent, in some cases), making the analogs suitable for clinical translation. One caveat of some of our analogs is that they are toxic to hair cells at high concentrations (9.98 μM) in zebrafish (Figures 3C,D). This toxicity was also observed when berbamine was tested in mice at concentrations greater than or equal to 30 μM (Kirkwood et al., 2017). However, analog toxicity was observed at much higher concentrations than the optimally protective concentration. For example, the OPC for BA-7 is 300-fold lower than the ototoxic concentration. Toxicity of berbamine and some of its analogs at higher concentrations may be due to the *para-*methylene phenol moiety that is common to many bisbenzylisoquinolines, which can lead to reactive intermediates (Tian and Zheng, 2017). Furthermore, many clinical drugs such as Amphetamine and Dapsone are toxic at high concentrations but highly effective at low concentrations (Schulz and Schmoldt, 2003). An advantage of using analogs with natural scaffolds is they can be readily modified to enhance desired characteristics such as physiological stability, solubility, and efficiency (Ji et al., 2009). Moving forward we plan to determine the pharmacokinetics of our lead analogs and further investigate the protective mechanism(s) of these leads in rodents as a prelude to clinical trials.

In summary, our study demonstrates that berbamine analogs with phenolic moieties robustly protect against aminoglycoside-induced hair cell death, primarily by attenuating aminoglycoside entry into hair cells, with some analogs also conferring uptake-independent protection. Several of the berbamine analogs described here were more potent than ORC-13661, a compound in clinical trials as an otoprotectant. The multiple modes of protection of some of our analogs also bolster their clinical use since there are likely multiple relevant targets, meaning that a single compound may confer protection *via* multiple mechanisms to increase protective efficacy.

Our former work confirmed that berbamine does not interfere with the antibacterial efficacy of aminoglycosides (Kruger et al., 2016) suggesting that berbamine analogs are not likely to interfere with clinical aminoglycoside use but that hypothesis remains to be tested. The zebrafish lateral line is an excellent pre-clinical tool for drug discovery and lead compound optimization for further translational work; future research will address the extent of analog protection in mammals. From our zebrafish analog screen, we identified several analogs, particularly BA-3, BA-4, and BA-7, as leads for future studies; these potent compounds (ED_50_ < 0.25 μM for neomycin) demonstrate protection from neomycin and gentamicin and strong GTTR uptake block (Figure 10). Our research provides more insight into hair cell pathology and aminoglycoside-induced hearing loss, yielding new approaches to preserve hearing.

## Data Availability Statement

The raw data supporting the conclusions of this article will be made available by the authors, without undue reservation.

## Ethics Statement

The animal study was reviewed and approved by Institutional Animal Care and Use Committee, Washington State University.

## Author Contributions

AH and AC conceived the concept, designed the bulk of the experiments and wrote the bulk of the manuscript. ON, JW and BB developed the analog synthesis strategy and made the analogs. KK designed, conducted, and analyzed the calcium imaging experiments. AH, GL and AC conducted all other experiments and analyzed the associated data. All authors contributed to writing and editing.

## Conflict of Interest

The authors declare that the research was conducted in the absence of any commercial or financial relationships that could be construed as a potential conflict of interest.

## Abbreviations

MET: mechanotransduction
^1^H NMR: nuclear magnetic resonance spectra for proton
TLC: thin-layer chromatography
EM: E2 embryo medium
dpf: days post-fertilization
DMSO: dimethylsulfoxide
DASPEI: 2-[4-(dimethylamino)styryl]-N-ethylpyridinium iodide
MS-222: Tricaine methanesulfonate
GTTR: gentamicin conjugated with Texas Red
OPC: optimal protective concentration
TRP channels: transient receptor potential channels
NMDA: N-methyl-D-aspartate
AMPA: α-amino-3-hydroxy-5-methyl-4-isoxazolepropionic acid
NF-κB: nuclear factor κ-light-chain-enhancer of activated B cells
JNK: c-Jun N-terminal kinase pathways
ERK: extracellular receptor kinase.
