# Supplementary material for: Berbamine Analogs Exhibit Differential Protective Effects From Aminoglycoside-Induced Hair Cell Death
Source: Front Cell Neurosci. 2020 Jul 29;14:234. doi: 10.3389/fncel.2020.00234 (PMC7403526; doi:10.3389/fncel.2020.00234)
Supplement: Supplementary file 1 [file Data_Sheet_1.PDF]

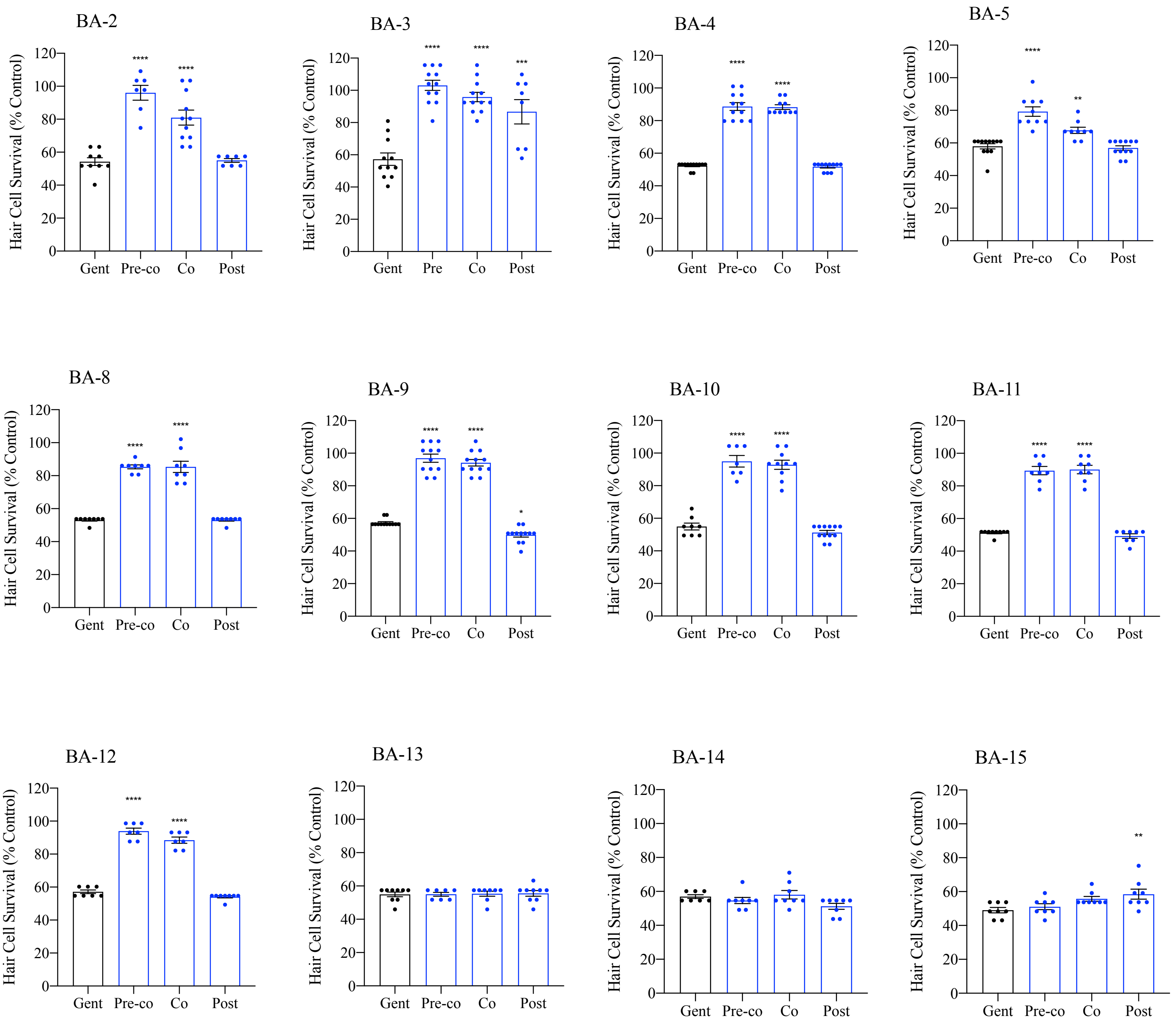

Supplementary Figure 1. Berbamine analogs display differential modes of protection from gentamicin. Given that berbamine analogs reduce GTTR uptake, analogs are predicted to confer protection when taken prior to or concurrently with gentamicin. Zebrafish were either pre-treated for 1 hour, co-treated for 30 minutes or post-treated for 5.5 hours with analog relative to 200 µM gentamicin exposure after the first hr. Analogs that showed protection in the pre-co and/or co- treatment groups are likely protecting hair cells by reducing aminoglycoside uptake via the MET channel (BA-2, BA-4, BA-5, BA-8, BA-9, BA-10, BA-11, BA-12). Analogs that show protection in all three treatment paradigms suggest that protection may occur not only protect hair cells by blocking the MET channel but also via other pathways (BA-3). Hair cells were assessed via DASPEI. Data were analyzed by one-way ANOVA, ****p<0.0001, ***p<0.001, N=10–12, bars are ± SEM. Refer to Supplementary Table 1 for statistics.

Supplementary Table 1. F-statistic and p-value for berbamine analog gentamicin washouts

Compound Name	ANOVA	p value by concentration (µM)
BA-1	$F_{3,44} = 74.07$<br>P<0.0001	Pre-co; ****<br>Co; ****
BA-2	$F_{3,30} = 27.36$<br>P<0.0001	Pre-co; ****<br>Co; ****
BA-3	$F_{3,39} = 24.58$<br>P<0.0001	Pre-co; ****<br>Co; ****<br>Post; ***
BA-4	$F_{3,41} = 195.6$<br>P<0.0001	Pre-co; ****<br>Co; ****
BA-5	$F_{3,39} = 28.63$<br>P<0.0001	Pre- co; ****<br>Co; **
BA-6	$F_{3,31} = 18.55$<br>P<0.0001	Per-co; ****<br>Co; ****<br>Post; **
BA-7	$F_{3,34} = 22.30$<br>P<0.0001	Pre-co; ****<br>Co; ****<br>Post; ****
BA-8	$F_{3,28} = 97.75$<br>P<0.0001	Pre-co; ****<br>Co; ****
BA-9	$F_{3,44} = 190.8$<br>P<0.0001	Pre-co; ****<br>Co; ****<br>Post; *
BA-10	$F_{3,33} = 102.8$<br>P<0.0001	Pre-co; ****<br>Co; ****
BA-11	$F_{3,29} = 143.1$<br>P<0.0001	Pre-co; ****<br>Co; ****
BA-12	$F_{3,25} = 211.0$<br>P<0.0001	Pre-co; ****<br>Co; ****
BA-13	$F_{3,29} = 0.04113$<br>P=0.9886	ns
BA-14	F 3,27 = 2.561<br>P=0.0758	ns
BA-15	F 3,28 = 4.528<br>P=0.0104	Post; **
BA-16	$F_{3,28} = 1.791$<br>P=0.1718	ns

****p<0.0001, ***p<0.001, **p<0.01, *p<0.05

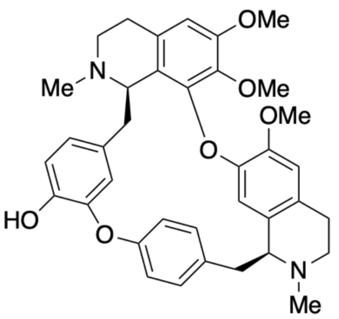

berbamine

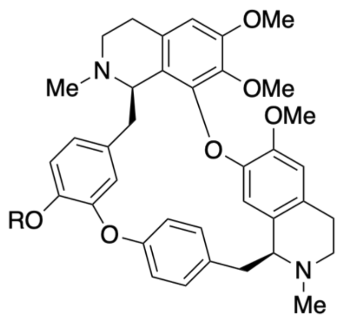

isotetrandrine; R = Me
E6 berbamine; R = 4-nitrophenacyl

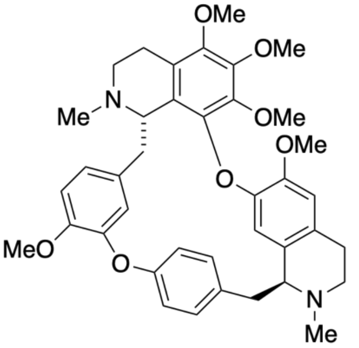

hernandezine

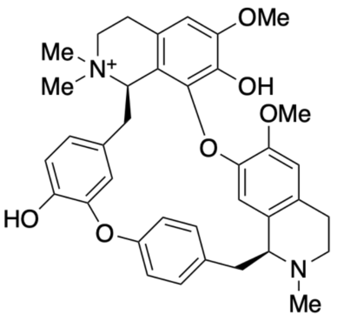

d-tubacurarine

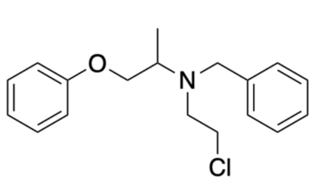

phenoxybenzamine

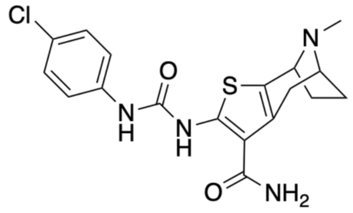

ORC-13661

Supplementary Figure 2. Berbamine-related analogs found in the original screen (Kruger et al. 2016) to protect zebrafish lateral line hair cells from aminoglycoside damage, as well as other published compounds found to have activity as otoprotectants.
